# A Randomized Trial with Two Hypocaloric Diets with Different Lipid Profiles and Effects on Serum Omentin-1 Levels in Obese Subjects

**DOI:** 10.1155/2022/6777283

**Published:** 2022-03-07

**Authors:** Daniel de Luis, Olatz Izaola, David Primo, Rocio Aller

**Affiliations:** Endocrinology and Nutrition Research Center, School of Medicine, Department of Endocrinology and Nutrition, Hospital Clinico Universitario, University of Valladolid, Valladolid, Spain

## Abstract

**Background:**

The effects of weight loss therapies on omentin-1 levels have been unclear, showing both elevations and decreases in circulating levels. The role of dietary fat might have an important role. The aim of our investigation was to evaluate the influence of weight decrease on omentin-1 levels after two different high-fat hypocaloric diets.

**Methods:**

319 Caucasian obese subjects were randomly allocated during 12 weeks (Diet M (high monounsaturated fat diet) vs. Diet P (high polyunsaturated fat diet)). The mean age was 47.2 ± 5.0 years (range: 26-64), and the mean body mass index (BMI) was 37.9 ± 4.1 kg/m^2^ (range: 30.6-39.8). Sex distribution was 237 females (74.7%) and 72 males (25.3%). Anthropometric and biochemical parameters were evaluated at basal and after both diets. SPSS 23.0 has been used to realize univariant and multivariant statistical analysis.

**Results:**

After both diets, BMI, weight, fat mass, waist circumference, systolic blood, LDL-cholesterol, insulin levels, and HOMA-IR decreased in a statistical way from basal values. These improvements were similar in both diets. After Diet P, omentin-1 levels increase (21.2 ± 9.1 ng/ml: *P* = 0.02), and after Diet M, this adipokine increases (47.1 ± 11.2 ng/ml: *P* = 0.02), too. The increase of omentin-1 with Diet M was statistically significantly higher than that after Diet P (*P* = 0.01). A multiple regression analyses adjusted by age and sex reported a statistical relation between BMI (kg/m^2^) and insulin (UI/L) with omentin-1 levels.

**Conclusions:**

Our study demonstrated a significant improvement on serum omentin-1 levels after weight loss secondary to both diets; in contrast, omentin-1 improvement was higher with Diet M than with Diet P.

## 1. Introduction

Adipokines are considered an important biomarkers indicative of metabolic status and health (1). Adipokines are produced from adipose tissue and act in an endocrine, paracrine, and autocrine way. These molecules have been related with obesity and a lot of comorbidities (2, 3). One of the least evaluated adipokines is omentin; the body of literature on this adipokine is still quite limited.

Omentin is a 34 kDa fat depot-specific molecule, which has been initially isolated from visceral omental adipocytes (4). Omentin-1 is the main circulating form; there are two homologous isoforms of this adipokine, omentin-1 and omentin-2 (5). Low levels of omentin-1 have been related with a worse metabolic status (6). In cross-sectional studies, levels omentin-1 correlated negatively with body weight, body mass index (BMI), waist circumference, and biochemical parameters (fasting insulin, HOMA-IR, and serum leptin levels) (7). These previous investigations have shown a positive correlation with adiponectin levels and high-density lipoprotein cholesterol (HDL-C) (7). Even an intervention study has shown that treatment with recombinant omentin-1 enhanced insulin-stimulated glucose uptake in adipose tissue (8).

Effective weight loss treatments that reduce adiposity are of great importance in order to decrease associated comorbidities of obesity and to improve the ratio of proinflammatory and anti-inflammatory adipokines (9). There are few studies that have evaluated the effect of weight loss with different strategies (hypocaloric diets, drugs, or surgical treatments) on omentin-1 levels. Besides, the effects of these weight loss therapies on omentin-1 levels have been unclear, showing both elevations and decreases in circulating levels. Studies with bariatric surgery have shown a tendency to decrease omentin-1 levels independently of the surgical technique, but at some point, with biphasic responses in omentin-1 levels (10, 11). Studies with dietary interventions have shown a moderate decrease (12–15) or an absence of response (16). Perhaps, the distribution of macronutrients of the diet and especially the dietary fats in the previous results might have a role (17). For example, consumption of a diet enriched with monounsaturated fatty acids tended to increase omentin-1 (17). Therefore, there are a few studies that exist evaluating the effect of diet on omentin levels, with contradictory results. In addition, the role of dietary fat has not been evaluated in randomized intervention studies; therefore, it is an area of interest in clinical research in obese patients. On the other hand, the alteration of lipid levels is common in the obese patient, due to the insulin resistance that these patients present from a very young age (18). As far as we know, there are no intervention trials evaluating the effect of two different high fat hypocaloric diets with different fatty acid profile on omentin-1 levels after a weight reduction intervention.

The objective of our study was to investigate the role of weight loss on omentin-1 concentrations and other parameters after two high fat different hypocaloric diets (high monounsaturated vs high polyunsaturated fat) in Caucasian obese subjects.

## 2. Materials and Methods

### 2.1. Subjects and Procedure

Three hundred and nineteen participants gave a signed informed consent prior to participation in the study, and they were randomized to two different diets; Diet P (enriched in polyunsaturated fatty acids) and Diet M (enriched in monounsaturated fatty acids). This study was conducted according to the guidelines laid down in the Declaration of Helsinki; the local ethics committee (HCUVA) approved all procedures involving patients. Two physicians and two dietitians were involved in the evaluation of these patients. The recruitment of patients was a consecutive method of sampling among subjects sent from Primary Care Physicians with obesity. Potential participants had the following inclusion criteria: body mass index ranged from 30 kg/m^2^ to 40 kg/m^2^, and an age ranged from 20 to 60 years. Potential participants were excluded if they reported one of the next situations: cardiovascular disease, diabetes mellitus, use of a weight-loss medication, use of a hypocaloric diet during the previous 6 months, and finally the use of drugs such as glucocorticoids, anti-inflammatory drugs, oral contraceptive pills, angiotensin receptor blockers, angiotensin converting enzyme inhibitors, fibrates, or statins.

Pre- and postintervention testing occurred before and after the 12 weeks of treatment with both diets. In both testing sessions, obese subjects reported to the laboratory after a 10-hour overnight fast. Following 20-minute rest, the next parameters were recorded: anthropometric parameters (weight, height, waist circumference, and total fat mass by bioimpedance) and blood pressure. After this rest time, a 21-gauge needle was used to collect a venous blood sample into a 15 ml vacutainer tube. The samples were centrifuged at 1500xg for 10 minutes; the serum was aliquoted into cryovials and stored at-40°C until batch analysis. Fasting glucose, C-reactive protein (CRP), insulin, insulin resistance as homeostasis model assessment (HOMA-IR), lipid profile (LDL-cholesterol, HDL-cholesterol, and plasma triglyceride concentration), and omentin-1 were determined in these samples.

### 2.2. Dietary Intervention

All obese subjects in this interventional study received individualized counseling. They were provided with sample menus, recipes, and list of resources to assist them with food cooking. Subjects were randomly allocated to one of the next two diets: Diet P (enriched in polyunsaturated fatty acids) and Diet M (enriched in monounsaturated fatty acids), restricting 500 daily calories to the usual intake. This caloric intake was calculated by subtracting 500 calories from the caloric intake obtained with the Harris-Benedict formula. All recruited patients received instructions to record their daily dietary intake for five nonconsecutive days at basal time and after 12 weeks of intervention. A dietitian assessed the adherence to the diet each 2 weeks by a phone call. Dietary registrations were analyzed using a specific software (Dietsource®, Ge, Swi) (19).

The target distribution of energy derived from macronutrients in the both diets was quite similar: Diet P (45.7% of carbohydrates, 34.4% of lipids, and 19.9% of proteins) and Diet M (46.6% of carbohydrates, 34.1% of lipids, and 19.2% of proteins). The recommended distribution of dietary fats in Diet P was 21.8% of saturated fats, 55.5% of monounsaturated fats, and 22.7% of polyunsaturated fats (7 g per day of w6 fatty acids, 2 g per day of w3 fatty acids and a ratio w6/w3 of 3.5). The recommended distribution of fats in Diet M was 21.7% of saturated fats, 67.5% of monounsaturated fats, and 10.8% of polyunsaturated fats.

The recommended physical activity consisted of an aerobic exercise at least three times per week (60 min each) such as running, walking, cycling, and swimming. All physical activities were registered with a self-reported questionnaire.

### 2.3. Biochemical Assays

The lipid profile (total cholesterol, HDL-cholesterol, and triglycerides) and the other molecules such as C-reactive protein (CRP), fasting glucose, and insulin were determined using an automated analyzer COBAS INTEGRA 400® (Roche Diagnostic, Montreal, Canada). LDL-cholesterol was determined using Friedewald formula (LDL‐cholesterol = total cholesterol − HDL‐cholesterol − triglycerides/5) (20). The homeostasis model assessment (HOMA-IR) was used to evaluate insulin resistance (glucose × insulin/22.5) (21). Omentin-1 was evaluated by ELISA (Biovendor Laboratory, Inc., Brno, Czech Republic) (RD191100200R) (22).

### 2.4. Adiposity Parameters and Blood Pressure

Height and weight were measured, using a mechanical beam scale with a height rod (SECA, 216, Brooklyn, NY). Body mass index was computed as body weight in kg/(height in m^2^). Waist circumferences were measured using a tape measure at the level of the umbilicus. The BIA was performed between 8:00 and 9:15 hours, after an overnight fast and after a time of 15 minutes in the supine position (23) (Akern, EFG, It). Blood pressure was determined three times after a 5-minute rest with a random zero mercury sphygmomanometer and averaged (Omron, LA, CA).

### 2.5. Statistical Analysis

Sample size was calculated to detect differences over 10 ng/ml in omentin-1 levels after weight loss with 90% power and 5% significance (*n* = 150 in each diet group) (9). The results were expressed as the mean ± standard deviation. Parametric variables with normal distribution were studied with two-tailed Student's *t*-test. Nonparametric variables were evaluated with the Mann–Whitney *U* test. Categorical variables were analyzed with the chi-square test. The statistical analysis used to evaluate the omentin-1 and diet interaction was a univariate ANCOVA. Correlation analysis was realized with Pearson's and Spearmen's test as needed. Multiple regression analysis (stepwise method) was used to analyze relationship of omentin-1 concentrations as a dependent variable. A *P* value < 0.05 was considered significant. SPSS version 23.0 has been used to realize statistical analysis.

## 3. Results

319 Caucasian obese subjects were recruited in the study. The mean age was 47.2 ± 5.0 years (range: 26-64), and the mean body mass index (BMI) was 37.9 ± 4.1 kg/m^2^ (range: 30.6-39.8). Sex distribution was 237 females (74.7%) and 72 males (25.3%).

In the 164 subjects (38 males and 126 females) treated with Diet P, basal dietary intakes showed the next intakes: calories of 1929.2 ± 239.1 kcal/day, carbohydrates of 253.2 ± 13.9 g/day (55.3% of calories), fats of 65.1 ± 9.2 g/day (25.2% of calories), and proteins of 86.9 ± 8.2 g/day (19.5% of calories). During the intervention, the patients reached the recommendations of Diet P with 1448 ± 216.1 kcal per day, 45.9% carbohydrates, 34.3% lipids, and 19.8% proteins. They followed the next fat distribution: 21.8% saturated fats, 55.3% monounsaturated fats, and 22.9% polyunsaturated fats (7 g per day of w6 fatty acids, 2 g per day of w3 fatty acids, and a w6/w3 ratio of 3.5).

In the 155 subjects (34 males vs 111 females) treated with Diet M, basal dietary intakes were shown: calories of 1938.9 ± 121.1 kcal/day, carbohydrates of 261.2 ± 21.1 g/day (56.1% of calories), fats of 81.9 ± 13.2 g/day (23.4% of calories), and proteins of 68.1 ± 8.0 g/day (20.5% of calories). During the intervention, the patients reached the recommendations of the Diet M, based on the consumption of virgin olive oil, reaching 1.442 ± 128.1 kcal per day with 46.0% carbohydrates, 34.4% fats, and 19.6% % protein. The fat distribution was 21.6% saturated fat, 68.4% monounsaturated fat, and 10.0% polyunsaturated fat.

After both high-fat hypocaloric diets, the below-mentioned parameters decreased significantly: body mass index, weight, fat mass, waist circumference, and systolic blood pressure (Table 1). These improvements were similar in both diets.

Similarly, after weight loss with both hypocaloric diets, total cholesterol, LDL-cholesterol, insulin levels, and HOMA-IR decreased in a statistical way from basal values (Table 2). These improvements were similar in both diets. After Diet P, omentin-1 levels increased (21.2 ± 9.1 ng/ml: *p* = 0.02) and after Diet M, the levels of this adipokine increased (47.1 ± 11.2 ng/ml: *P* = 0.02), too. The improvement in the levels of omentin-1 with Diet M was higher than Diet P (*P* = 0.01).

Basal and posttreatment correlation analysis (Table 3) reported a positive association of omentin-1 basal levels with age and a negative correlation with BMI and insulin. After Diet M, in a multiple linear regression analyses adjusted by age and sex, BMI kg/m^2^ (beta: -0.25; 95% CI: -6.80-0.12) and insulin UI/L (beta: -0.31; 95% CI: -5.90-0.21) were two independent contributors to circulating basal omentin-1. The second multiple regression analyses after weight loss with Diet M adjusted by age and sex showed the same statistical association of BMI kg/m^2^ (beta: -0.23; 95% CI: -7.10-0.10) and insulin UI/L (beta: -0.29; 95% CI: -7.01-0.13) with posttreatment omentin-1 levels.

The same analysis was realized with Diet P. The multiple linear regression analyses before diet adjusted by age and sex reported BMI kg/m^2^ (beta: -0.19; 95% CI: -7.8-0.09) and insulin UI/L (beta: -0.21; 95% CI: -4.90-0.31) as two independent contributors to serum basal omentin-1. The second multiple regression analyses after weight loss with Diet P adjusted by age and sex showed a statistical association between BMI kg/m^2^ (beta: -0.18; 95% CI: -7.30-0.08) and posttreatment omentin-1 levels.

## 4. Discussion

To our knowledge, this is the first study to examine the effects of two different high-fat hypocaloric diets with different dietary fat profiles on serum omentin-1 levels. This randomized trial showed that energy restriction intervention either high-monounsaturated fat or high-polyunsaturated fat improved adiposity parameters, insulin, HOMA-IR, LDL-cholesterol, and omentin-1 levels. High monounsaturated fat diet increased omentin-1 levels more than a diet high in polyunsaturated fats.

To date scarce studies have evaluated the effects of weight loss secondary to dietary interventions on serum omentin-1 levels. Moreno-Navarrete et al. (12) reported that omentin-1 levels increase after a hypocaloric diet providing an energy restriction of 500-1000 kcal per day during 4 months. The percentage of macronutrient in this intervention was 16%, 30% and 54% of energy requirements from protein, fat, and carbohydrates, respectively. This diet had less dietary fat than the diet of our study, but they did not describe the percentage and type of unsaturated fats. Later, Lesńa et al. (13) observed that omentin-1 levels were unchanged during an intervention with a standard hypocaloric diet of 1 month and increased only after 12 months. Moreno-Navarrete et al.(12) reported an increment in omentin-1 levels after weight loss secondary to a hypocaloric diet with Mediterranean pattern. This diet had 53% of carbohydrates, 26% of lipids, and 21% of proteins, with a 50.5% of monounsaturated fatty acids. The response of omentin-1 in other study was different (13); different amount to dietary fats were included in both diets (36% vs 26%). In another interventional study (24), a vitamin D-fortified low-fat yogurt for 3 months decreased adiposity parameters and increased omentin-1 levels in postmenopausal females. In studies with children, an increase in omentin-1 levels has also been shown after a hypocaloric diet. Siegrist et al. (25) reported effects of a short-term weight reduction (1 month) on BMI and omentin-1 levels in children. The same results were observed by Zhang et al. (26) in a long-term lifestyle intervention during 6 months in obese children (aged 7-18 years) with metabolic syndrome.

Moreover, short-term study of 2 weeks (11) with a very low caloric diet had not significant effect on omentin-1 levels. The same lack of effect on omentin-1 levels was reported by Graff et al. (16) with a Paleolithic diet for 3 months. Finally, supplementation with vitamin D in a hypocaloric diet in obese adults for 2 months also failed to modify omentin-1 levels (27). Nevertheless, the increase in omentin-1 after the great weight losses demonstrated after bariatric surgery is more consistent throughout the different studies in the literature (27–30).

These contradictory results in the modification of omentin-1 levels might be related to multiple factors: for example, the different age range and ethnicity of the obese patients recruited in the studies, the body mass index range of the patients, the intervention time (range 15 days to 12 months), the percentage of reduction in the previous caloric intake, and the distribution of the macronutrients, particularly different types of fatty acids. In our design, the main type of fatty acids was unsaturated in Diet M and Diet P, too. The direct effect of dietary fatty acids has been demonstrated by Kabiri et al. (17). In our design, both diets produced an increase in the levels of omentin-1; however, this increase was greater in the patients who received the diet rich in monounsaturated fats with the same amount of weight loss than the diet rich in polyunsaturated fats. This allows us to hypothesize a direct effect of the type of dietary fat on omentin-1 levels.

Our data showed an inverse correlation of circulating omentin-1 levels with body mass index and HOMA-IR. This fact has been reported previously in the literature (12), too. This relationship has made it possible to demonstrate an association of hyperinsulinemia with circulating levels of omentin-1 and inhibits its secretion (31). Therefore, we could hypothesize that weight loss increases omentin-1 levels via increasing insulin sensitivity. However, in our study, both diets decreased hyperinsulinemia and body weight, but an increase of omentin-1 levels higher in patients with Diet M than Diet P has been observed. Therefore, some changes in omentin-1 might be attributable to the effects of dietary fatty acids on their gene expression or other unknown effect. As we have previously commented (17), some authors have demonstrated that consumption of olive oil-rich diet increased omentin-1 levels. Some research groups are evaluating therapeutic options to raise omentin-1 levels with metformin (32) and recombinant leptin (33), too. Therefore, the current findings of the effect of the type of unsaturated fats on omentin-1 levels may be important as a therapeutic option to improve its levels and metabolic benefits. However, to interpret our results, it is important to consider the systemic inflammation that obese patients present. For example, Nod-like receptor family pyrin domain-containing protein-3 (NLRP3) complex inflammasome has potentially been shown to play an important role in the development of diabetes in these patients (34), and some studies have demonstrated that a soluble urokinase-type plasminogen activator receptor (suPAR) plays an essential function in leukocytes and endothelial homeostasis and, therefore, in the development of coronary heart disease (35). Systemic inflammation in obese subjects has a main role in cardiovascular disease and metabolic parameters related with adipokines (36).

One limitation of our study was that the compliance and monitoring of the diet were carried out with a self-administered food questionnaire. Second, the correlations and multivariant results cannot be interpreted as causative. Third, other limitation is the absence of a control group, which could confirm that the results of the present investigation were due to the dietary change. Fourth, the lack of other adipokines such as vaspin, visfatin, leptin, or resistin or even mRNA expression of omentin-1 is an important limitation in order to explain pathophysiological mechanisms. Fifthly, the small sample size can produce a lack of statistical power. Finally, our sample was a sample of adult obese patients and this sample was no necessarily representative of the general population.

In conclusion, the data from our study demonstrated a significant increase on serum omentin-1 levels after weight loss secondary to both high-fat hypocaloric diets. Additionally, omentin-1 improvement was higher with monounsaturated fatty acid-enriched diet than polyunsaturated fatty acid-enriched diet. For this reason, in the diets of obese patients, in addition to caloric restriction, it is necessary to take into account the type of fat that the diet contains. Monounsaturated fat shows greater benefits on exomentian-1 levels, which in turn may have more long-term benefits in relation to complications or the inflammatory state of the obese patient.

## Figures and Tables

**Table 1:** Changes in adiposity parameters and blood pressure (mean ± SD).

Parameters	Diet M (*n* = 164)		Diet P (*n* = 155)		
	Basal	12 weeks	Basal	12 weeks	*P* value Time intervention Diet M Basal values Time intervention Diet P Post 12 weeks values
BMI	37.9 ± 2.1	36.2 ± 3.2^∗^	38.0 ± 3.3	35.6 ± 3.9^∗^	*P* = 0.03 *P* = 0.31 *P* = 0.02 *P* = 0.34
Weight (kg)	97.5 ± 1.2	93.2 ± 2.1^$^	95.2 ± 3.1	91.6 ± 1.3^$^	*P* = 0.03 *P* = 0.41 *P* = 0.02 *P* = 0.55
Fat mass (kg)	40.6 ± 1.2	37.3 ± 1.3^#^	38.9 ± 1.1	35.4 ± 2.0^#^	*P* = 0.03 *P* = 0.46 *P* = 0.03 *P* = 0.47
WC (cm)	113.5 ± 3.1	109.5 ± 3.1^&^	112.5 ± 5.1	109.1 ± 3.1^&^	*P* = 0.01 *P* = 0.38 *P* = 0.02 *P* = 0.48
SBP (mmHg)	127.1 ± 2.2	123.6 ± 1.9^∗∗^	126.9 ± 7.2	122.1 ± 5.0^∗∗^	*P* = 0.02 *P* = 0.31 *P* = 0.03 *P* = 0.40
DBP (mmHg)	81.2 ± 3.0	79.8 ± 4.2	81.8 ± 6.1	80.2 ± 3.1	*P* = 0.41 *P* = 0.69 *P* = 0.62 *P* = 0.67

BMI: body mass index; WC: waist circumference; SBP: systolic blood pressure; DBP: diastolic blood pressure. Statistical differences: *P* < 0.05, in each group diet (^∗^BMI, ^$^weight, ^#^fat mass, ^&^WC, and ^∗∗^SBP). First *P*, significant difference in Diet M dietary intervention at 3 months, second *P*, statistical significance between baseline values of the two diets, third *P*, significant difference in Diet P dietary intervention at 12 weeks, and fourth *P*, statistical significance between values at 12 weeks of the two diets.

**Table 2:** Biochemical parameters (mean ± SD).

Parameters	Diet M (*n* = 164)		Diet P (*n* = 155)		
	Basal	12 weeks	Basal	12 weeks	*P* value Time intervention Diet M Basal values Time intervention Diet P Post 12 weeks values Comparación 3 meses
Glucose (mg/dl)	101.1 ± 10.1	99.7 ± 7.0	103.6 ± 8.0	99.1 ± 7.0	*P* = 0.11 *P* = 0.54 *P* = 0.12 *P* = 0.43
Cholesterol total (mg/dl)	208.9 ± 5.7	199.2 ± 7.2^$^	204.2 ± 6.1	194.7 ± 4.2^$^	*P* = 0.03 *P* = 0.51 *P* = 0.04 *P* = 0.31
LDL-cholesterol (mg/dl)	128.9 ± 8.1	123.3 ± 6.2^#^	125.3 ± 7.1	121.1 ± 3.2^#^	*P* = 0.01 *P* = 0.44 *P* = 0.02 *P* = 0.39
HDL-cholesterol (mg/dl)	53.8 ± 1.6	52.2 ± 1.9	50.9 ± 2.0	50.3 ± 1.3	*P* = 0.03 *P* = 0.04 *P* = 0.59 *P* = 0.03
Triglycerides (mg/dl)	124.2 ± 11.0	122.8 ± 9.2	133.2 ± 8.2	123.1 ± 5.1	*P* = 0.12 *P* = 0.51 *P* = 0.20 *P* = 0.47
Insulin (mUI/l)	13.1 ± 2.1	11.1 ± 1.2^&^	13.1 ± 2.2	11.7 ± 2.0^&^	*P* = 0.03 *P* = 0.40 *P* = 0.03 *P* = 0.43
HOMA-IR	3.3 ± 1.2	2.7 ± 0.9^∗∗^	3.5 ± 0.9	2.9 ± 1.1^∗∗^	*P* = 0.01 *P* = 0.37 *P* = 0.02 *P* = 0.41
CRP	4.3 ± 1.1	4.1 ± 1.2	4.6 ± 2.0	4.5 ± 2.9	*P* = 0.21 *P* = 0.34 *P* = 0.33 *P* = 0.42
Omentin-1 (ng/dl)	572.9 ± 14.2	629.6 ± 13.2^#^	568.47 ± 13.1	589.4 ± 8.3^#^	*P* = 0.02 *P* = 0.02 *P* = 0.13 *P* = 0.03

HOMA-IR: homeostasis model assessment; CRP: C reactive protein. Statistical differences: *P* < 0.05, in each group diet (^$^total cholesterol, ^#^LDL-cholesterol, ^∗^HDL-cholesterol, ^&^insulin, and ^∗∗^HOMA-IR). First *P*, significant difference in Diet M dietary intervention at 3 months, second *P*, statistical significance between baseline values of the two diets, third *P*, significant difference in Diet P dietary intervention at 12 weeks, and fourth *P*, statistical significance between values at 12 weeks of the two diets.

**Table 3:** Correlation analysis between omentin-1 and basal-postintervention parameters.

Parameters	Basal Diet M	Posttreatment Diet M	Basal Diet P	Posttreatment Diet P
Age	*r* = 0.46, *P* = 0.001	*r* = 0.44, *P* = 0.001	*r* = 0.41, *P* = 0.01	*r* = 0.33, *P* = 0.03
BMI	*r* = −0.36, *P* = 0.002	*r* = −0.33, *P* = 0.01	*r* = −0.240, *P* = 0.04	*r* = −0.35, *P* = 0.02
Insulin	*r* = −0.48, *P* = 0.001	*r* = −0.38, *P* = 0.001	*r* = −0.370, *P* = 0.02	*r* = −0.36, *P* = 0.01

BMI: body mass index.

## Data Availability

All data generated or analyzed during this study are included in this article. Further enquiries can be directed to the corresponding author.
